# Epidemiological characteristics, distribution of axial length, and prevalence of ocular abnormalities associated with congenital cataract in Northwest China: a 16-year cross-sectional study (2008–2023)

**DOI:** 10.3389/fmed.2025.1617601

**Published:** 2025-09-05

**Authors:** Mengmei He, Min Gong, Guorui Dou, Jian Zhou, Xu Hou

**Affiliations:** Department of Ophthalmology, Eye Institute of Chinese PLA, Xijing Hospital, Fourth Military Medical University, Xi’an, Shaanxi, China

**Keywords:** congenital cataract, epidemiology, axial length, ocular abnormalities, Northwest China

## Abstract

**Background:**

We aimed to identify epidemiological characteristics, distribution of axial length and ocular abnormalities associated with congenital cataract in Northwest China.

**Methods:**

This observational cross-sectional study evaluated patients with congenital cataracts who underwent surgery between 2008 and 2023. Patient characteristics were compared among different age groups and hospitalization years.

**Results:**

A total of 527 patients (527 eyes) underwent cataract surgeries. Of these, 323 patients (61.29%) resided in rural areas and 204 (38.71%) in urban regions. Rural patients exhibited a higher median age at surgery (36[6,72] months) compared to urban counterparts (23.5[4,64] months), with a significant difference between them (*Z =* −2.543, *p* = 0.011). Median surgical age differs significantly across hospitalization year subgroups (*χ*^2^ = 40.636, *p* < 0.001). Axial length demonstrated accelerated growth prior to 2 years of age, with statistically significant interocular differences in unilateral cases below 24 months (*p* < 0.05). Congenital cataract cases were predominantly diagnosed between 2 and 6 years (32.26%). Age distribution patterns significantly varied across hospitalization periods (*χ*^2^ = 70.394, *p* < 0.001). Ocular abnormalities alone were present in 293 cases (55.60%), while 40 patients (7.59%) exhibited concurrent systemic abnormalities. Amblyopia emerged as the most frequent ocular comorbidity (23.34%).

**Conclusion:**

Rural patients constituted a higher proportion of congenital cataract. The median surgical age demonstrated a significant difference across hospitalization year subgroups. Axial growth patterns showed significant correlations with surgical timing, sex, and laterality. These findings indicate enhanced parental awareness of pediatric ocular health and provide an evidence-based rationale for early intervention strategies in congenital cataracts in Northwest China.

## 1 Introduction

Congenital cataract (CC) is a leading cause of treatable blindness in children worldwide, with significant ethnic and regional variations (1). While its prevalence is reported as 3–4 per 10,000 births in Western countries and 5 per 10,000 in China overall (2, 3), comprehensive epidemiological data specific to Northwest China, a region characterized by socioeconomic disparities and limited healthcare access, remain notably absent in existing literature. This gap is critical, as delayed diagnosis and suboptimal management in resource-limited settings may exacerbate visual disability.

The surgical age was defined as the primary endpoint. We hypothesize that the patients’ age at surgery showed a disparity throughout the study period from 2008 to 2023 (2008–2011, 2012–2015, 2016–2019, and 2020–2023). The exploratory endpoints included axial length (AL), laterality, sex, place of residence, surgical method, and associated ocular and systemic abnormalities. Understanding the epidemiology and clinical features of CC is clinically imperative: early identification of regional risk factors can guide targeted infant screening programs, while characterizing surgical delays and complications informs optimized treatment protocols to prevent irreversible amblyopia.

Therefore, this study aims to describe the epidemiological characteristics, surgical timelines, ocular biometric parameters, and associated complications of CC in Northwest China. We retrospectively analyzed 527 patients undergoing cataract surgeries at Xijing Hospital from 2008 to 2023 to address this unmet need, providing foundational data for public health interventions in underserved populations.

## 2 Methods

### 2.1 Study design and subjects

This study was carried out at Xijing Hospital of the Fourth Military Medical University in Xi’an, China. This hospital is a tertiary referral center in Northwest China, and is currently the primary hospital for CC treatment. All surgical patients in our hospital prospectively sign an informed consent form prior to surgery by their legal guardians, which explicitly authorizes the future use of their de-identified medical data for research purposes. These pre-existing consents complied with national and international guidelines (e.g., Helsinki Declaration), ensuring robust de-identification protocols. Those patients who agree to the surgery but do not consent to the use of their data for analysis will indicate this when signing the consent form, and their medical records will be excluded from future medical research. This hospital-based, observational study was approved by the institutional ethics committee of Xijing Hospital (Approval number: KY20212051) and was conducted in accordance with the principles of the Declaration of Helsinki. Patient information was anonymized and de-identified before this analysis.

### 2.2 Inclusion and exclusion criteria

This study collected the data of patients who were hospitalized for CC from 2008 to 2023. The inclusion criteria were: (1) A clear diagnosis of CC (4, 5) during the period from 2008 to 2023; (2) Written informed consent were provided by legal guardians for the use of medical data; (3) Patients who underwent their first cataract surgery during the study period. The exclusion criteria were: (1) Patients with congenital intrauterine infection cataract (such as rubella cataract), complicated cataract (such as uveitis cataract), metabolic cataract (such as tetany cataract), and traumatic cataract; (2) Patients with a history of other eye surgeries, trauma, infection, cough or other systemic diseases likely to affect the AL examination and surgical procedure; (3) Patients with incomplete medical records.

### 2.3 Patient subgroups

Data were obtained from the hospital medical records by two independent researchers. The proportion of CC among all cataracts, age at surgery, sex, place of residence, laterality, AL, surgical method, and associated ocular and systemic abnormalities were analyzed. To explore the characteristics of AL in different age subgroups and age distribution in different periods, we divided the study population into six age subgroups. Age subgroups were defined by visual development critical periods: 0–6M (≤6 months: critical period for visual development in infants and young children), 6M–2Y (>6 months and ≤2 years: the period of establishing binocular visual function), 2Y–6Y (>2 years and ≤6 years: coincide with the initiation of preschool vision screening programs or parental observation of visual behaviors, leading to diagnosis; Represents a common “window” for delayed diagnosis beyond infancy), 6Y–2Y (>6 years and ≤12 years) and 12Y–18Y (>12 years and ≤18 years): segregates pre-pubertal vs. adolescent phases where ocular growth rates and surgical compliance differ significantly, >18Y (>18 years: represents adult patients with stable ocular anatomy). To ensure statistical robustness and balanced sample sizes for temporal trend analysis, the study population was divided into four subgroups based on hospitalization period: 2008–2011, 2012–2015, 2016–2019, and 2020–2023.

### 2.4 Statistical analysis

Measurement data following normal distributions, such as axial length, are presented as mean ± standard deviation (SD), whereas non-normally distributed measures like surgical age are reported as median and interquartile range (IQR). Descriptive data are presented as numbers and percentages. The Kolmogorov–Smirnov test was used to evaluate the normality of the distribution of variables (Supplementary Table S1). Significant differences between the two groups were determined using the Student’s *t*-test or Wilcoxon’s rank-sum test (Mann–Whitney *U* test). Meanwhile, a one-way analysis of variance with the Kruskal–Wallis test was performed to compare data among multiple groups. Student’s *t*-tests were used to compare the axial length between boys and girls in different age groups and adjusted for multiple testing with False Discovery Rate (FDR) correction. The difference in AL between the affected and fellow eyes in patients with unilateral CC in different age groups was tested using a paired t-test and adjusted for multiple testing with FDR correction. The Jonckheere-Terpstra test was used to evaluate the correlation between the age of surgery and the year of hospitalization. The Chi-square test was used to compare the frequency and composition ratios between different groups. Pearson’s correlation coefficients and regression analyses were performed to analyze the relationship between age at surgery and AL. 95% Confidence Interval (CI) was determined using Agresti-Coull. All statistical analyses were performed using the Statistical Package for the Social Sciences (SPSS 26.0; IBM Corp., Armonk, NY, United States). Statistical significance was defined as a two-sided *p*-value of <0.05.

## 3 Results

### 3.1 Patient characteristics

**Figure 1:**
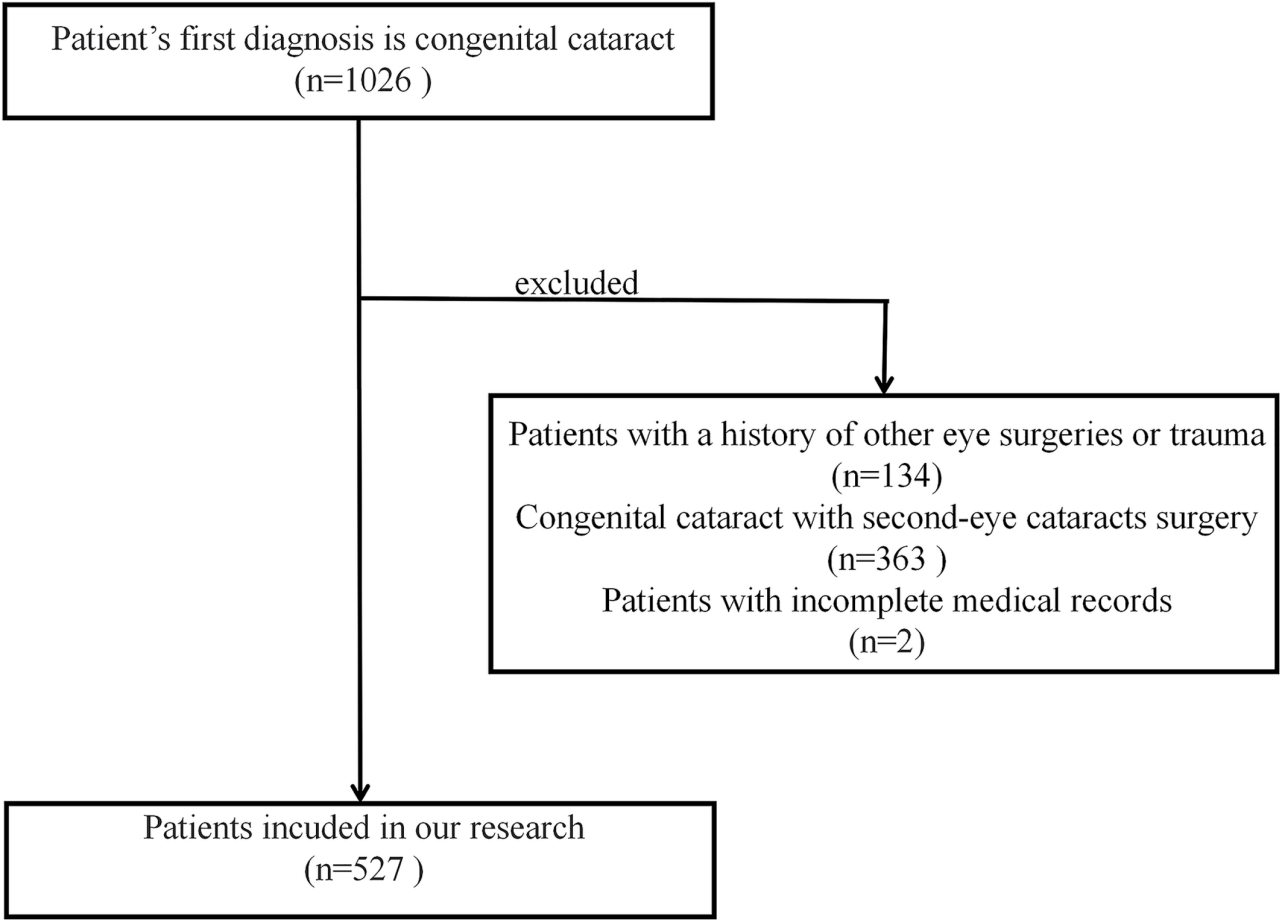
A flow diagram summarizing participant selection and inclusion.

A total of 527 patients (280 males and 247 females) with CC were enrolled in this study (Figure 1 and Supplementary Table S2). The gender distribution did not differ significantly across hospitalization years (*χ*^2^ = 15.067, *p* > 0.05) or age groups (*χ*^2^ = 4.379, *p* > 0.05). The study comprised 527 patients, with rural residents representing 61.29% (*n* = 323) and urban residents 38.71% (*n* = 204). Rural patients exhibited a higher median age at surgery (36[6,72] months) compared to urban counterparts (23.5[4,64] months), with a significant difference between them (*Z =* −2.543, *p* = 0.011).

**Figure 2:**
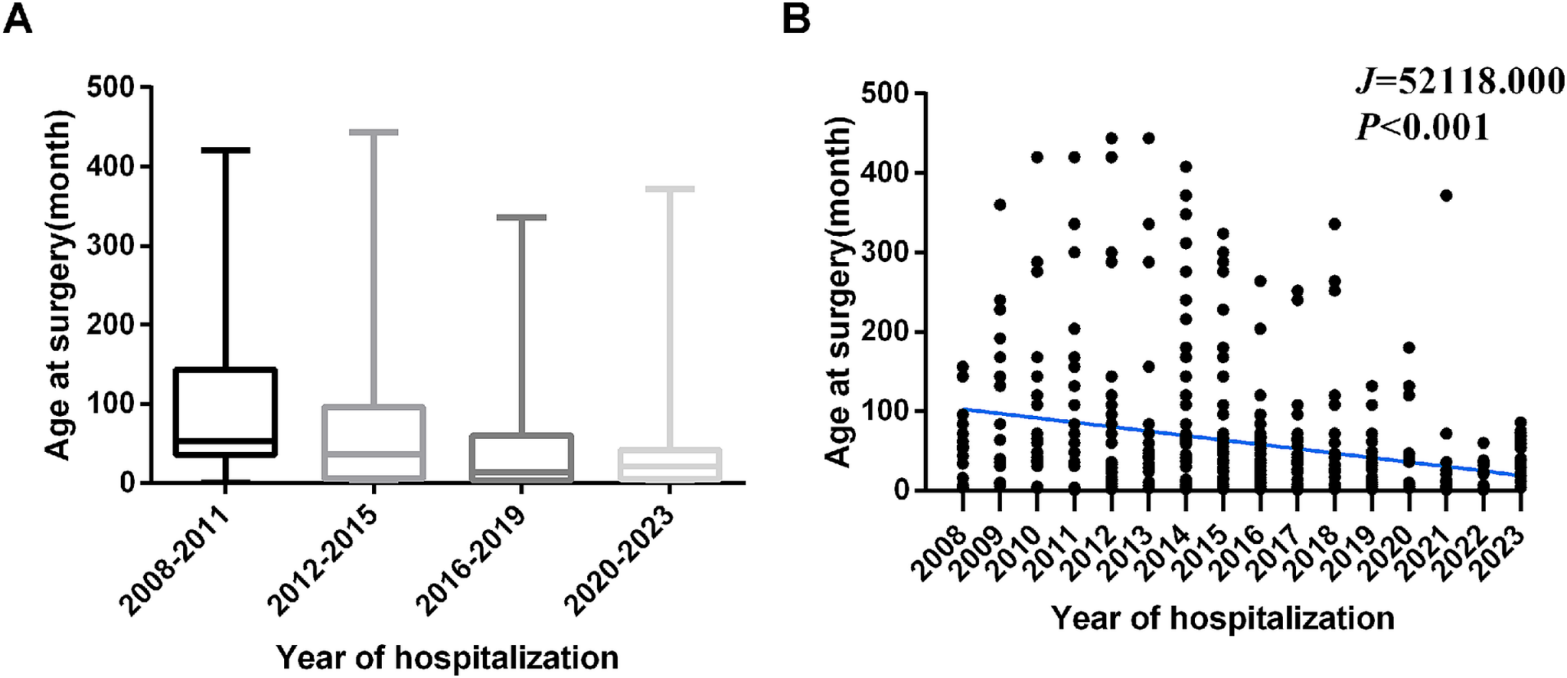
The median surgical age differs significantly across hospitalization year subgroups. **(A)** The median age at surgery in hospitalization period. **(B)** Trends in the age at surgery by hospitalization period.

**Figure 3:**
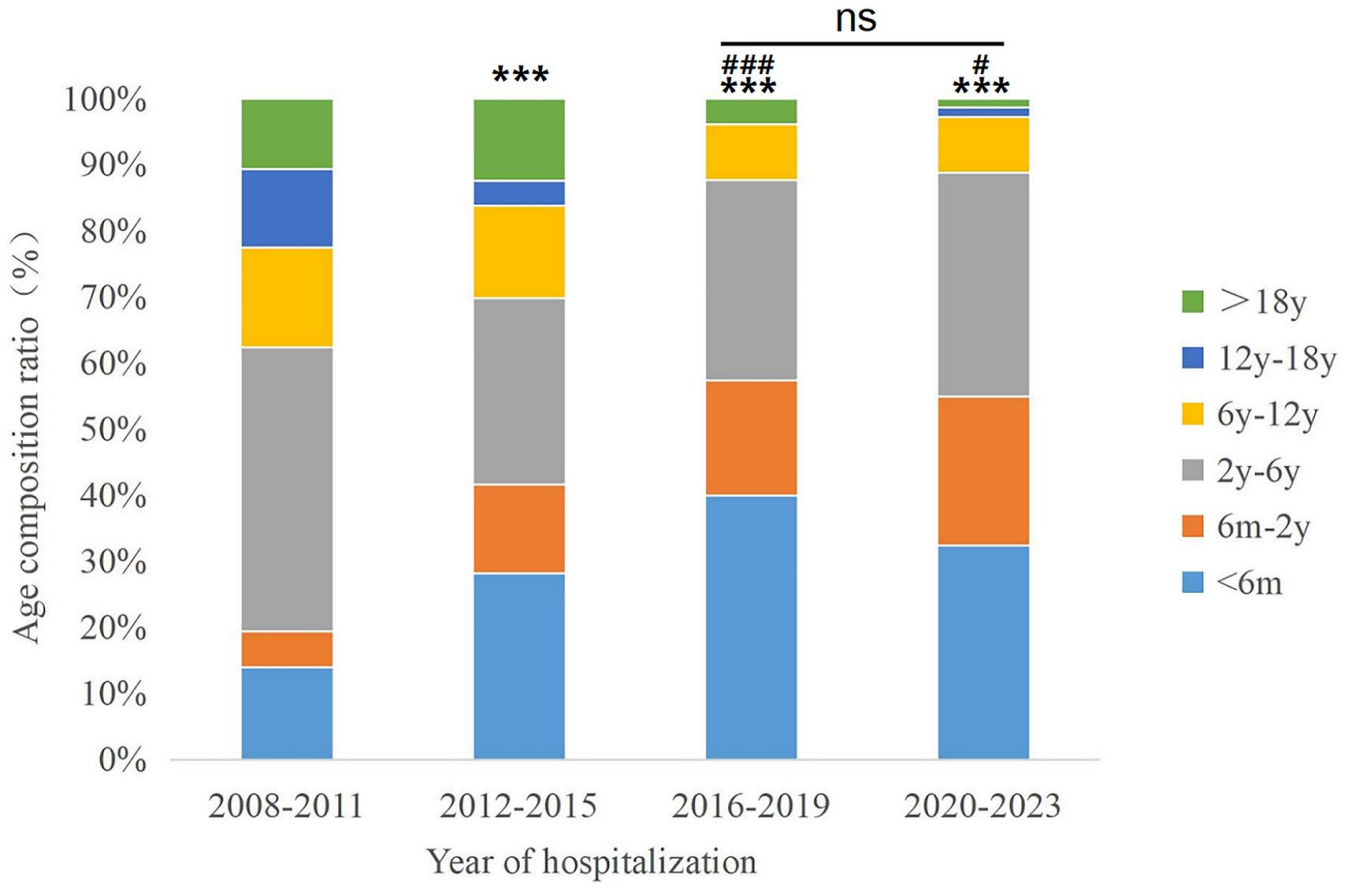
Age composition ratio of patients with congenital cataract across hospitalization periods. Ns, non-significant (*p* > 0.05), ^***^P < 0.001 vs. 2008–2011 group; ^#^*p* < 0.05, ^###^*p* < 0.001 vs. 2012–2015 group.

The median age at surgery across the entire population was 34.00[5.00,72.00] months. Median surgical age differs significantly across hospitalization year subgroups (*χ*^2^ = 40.636, *p* < 0.001). Significant negative correlation was found between surgical age and year of hospitalization (*J =* 52118.000, *p* < 0.001), indicating a progressive downward trend in surgical age over the study period from 2008 to 2023 (Figures 2A,B). Age distribution analysis revealed a predominance of patients aged 2–6 years (32.26%, 170/527), contrasting with limited representation of adolescents aged 12–18 years (7.59%, 40/527, Table 1). There were significant differences in the age composition ratio between different periods (*χ*^2^ = 70.394, *p* < 0.001, Figure 3), characterized by a marked increase in infants undergoing surgery ≤2 years of age and a concurrent decline in patients >12 years.

**Table 1:** Age distribution of patients with congenital cataracts.

Age	*N*	Proportion (%)	Median age [IQR] (month)
0-6M	159	30.17%	3[3,5]
6M–2Y	77	14.61%	12[8,16]
2Y–6Y	170	32.26%	47[36,64]
6Y–12Y	61	11.57%	108[84,120]
12Y–18Y	20	3.80%	180[168,201]
>18Y	40	7.59%	300[276,345]

N, number of patients; IQR, interquartile range.

The surgical population comprised two distinct management strategies: primary intraocular lens (IOL) implantation was performed in 271 patients, with median surgical age of 72[42,120] months, while 256 patients with median surgical age of 5[3,11] months underwent a staged approach involving initial cataract extraction without IOL implantation, followed by secondary IOL placement during subsequent hospitalization. There was a statistically significant difference in the age of surgery between the two groups (*Z =* −17.649, *p* < 0.001).

### 3.2 Axial length distribution

**Figure 4:**
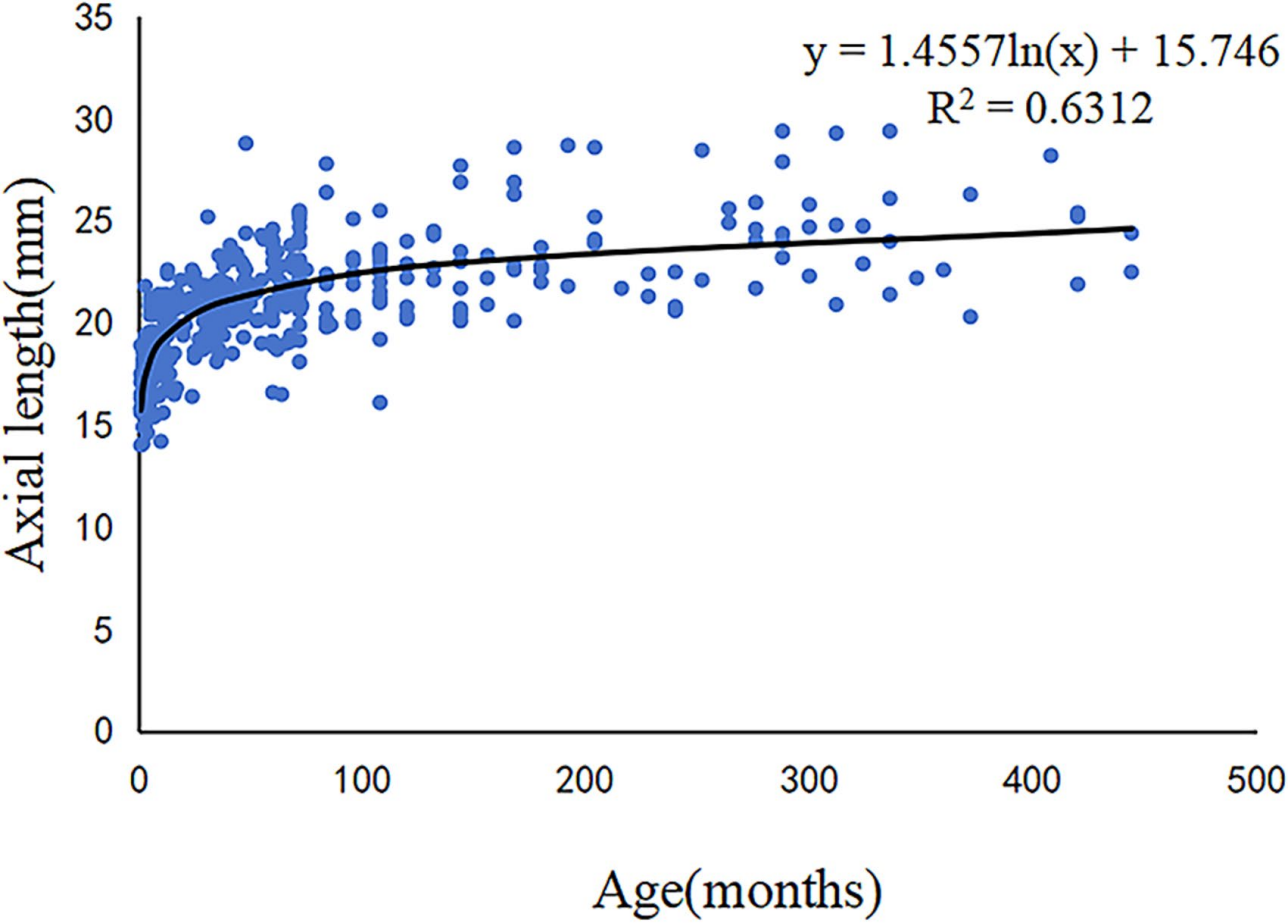
Scatter plots and fitting curve of the correlation between axial length and age in patients with congenital cataract.

A logarithmic correlation between AL and age was identified at the time of surgery (*R^2^* = 0.6312, *p* < 0.001; Figure 4), with distinct growth phases observed across age groups. A faster trend of AL elongation was observed before the age of 2 years, whereas, AL changes tended to stabilize after the age of 6 years. Among 527 CC patients, 33.78% (178/527) presented with unilateral involvement, showing a slight predominance of left-eye involvement (18.22%, 96/527) over right-eye cases (15.56%, 82/527). Sex-based disparities in AL were prominent in bilateral CC patients. Males aged 6M–2Y, 2Y–6Y demonstrated significantly longer AL compared to females (*p* < 0.05, Table 2). Conversely, no sex-related AL differences were observed in unilateral CC populations across all age groups (*p* > 0.05, Table 2). There was a significant difference in AL between the affected and fellow eyes in patients with unilateral CC aged 6M–2Y (*t* = −3.056, *p* = 0.030, Table 3).

**Table 2:** Comparison of the axial length between boys and girls with adjustment for age and laterality of congenital cataract.

	0–6M	6M–2Y	2Y–6Y	6Y–12Y	12Y–18Y	>18Y
Bilateral						
Boys AL (mm)	17.55 ± 1.41	20.24 ± 1.81	21.80 ± 1.68	22.03 ± 1.92	24.70 ± 2.74	25.41 ± 2.55
	*n* = 77	*n* = 26	*n* = 58	*n* = 16	*n* = 4	*n* = 15
Girls AL (mm)	17.25 ± 1.17	18.40 ± 1.74	20.51 ± 1.85	21.55 ± 1.99	24.36 ± 2.71	23.39 ± 1.67
	*n* = 53	*n* = 26	*n* = 42	*n* = 17	*n* = 5	*n* = 10
*t*	1.279	3.744	3.622	0.703	0.186	0.279
*p*	0.3045	0.003*	0.003*	0.5856	0.857	0.076
Unilateral						
Boys AL (mm)	17.90 ± 1.23	19.10 ± 1.29	21.30 ± 1.93	22.58 ± 1.83	22.73 ± 1.89	25.04 ± 2.95
	*n* = 18	*n* = 13	*n* = 28	*n* = 13	*n* = 7	*n* = 5
Girls AL (mm)	18.13 ± 1.54	18.90 ± 1.74	21.56 ± 1.73	22.88 ± 2.38	24.78 ± 3.64	22.89 ± 2.41
	*n* = 11	*n* = 12	*n* = 42	*n* = 15	*n* = 4	*n* = 10
*t*	−0.457	0.142	−0.573	−0.367	−1.253	1.521
*p*	0.750	0.750	0.750	0.750	0.726	0.726

AL: Axial length; *p < 0.05.

**Table 3:** Difference in axial length between the affected and fellow eye in patients with unilateral congenital cataract.

Age	N	Affected eye (mm)	Fellow eye (mm)	*t*	*p*
0-6M	29	18.52 ± 1.05	17.99 ± 1.33	−2.377	0.075
6M–2Y	25	19.76 ± 1.06	19.00 ± 1.49	−3.056	0.030*
2Y–6Y	70	21.45 ± 1.80	21.38 ± 0.95	0.424	0.808
6Y–12Y	28	22.72 ± 2.10	22.34 ± 1.14	1.019	0.634
12Y–18Y	11	23.48 ± 2.44	22.65 ± 3.28	0.699	0.743
>18Y	12	23.60 ± 2.70	23.50 ± 2.47	0.164	0.872

*P < 0.05.

### 3.3 Proportion of associated systemic and ocular abnormalities

**Figure 5:**
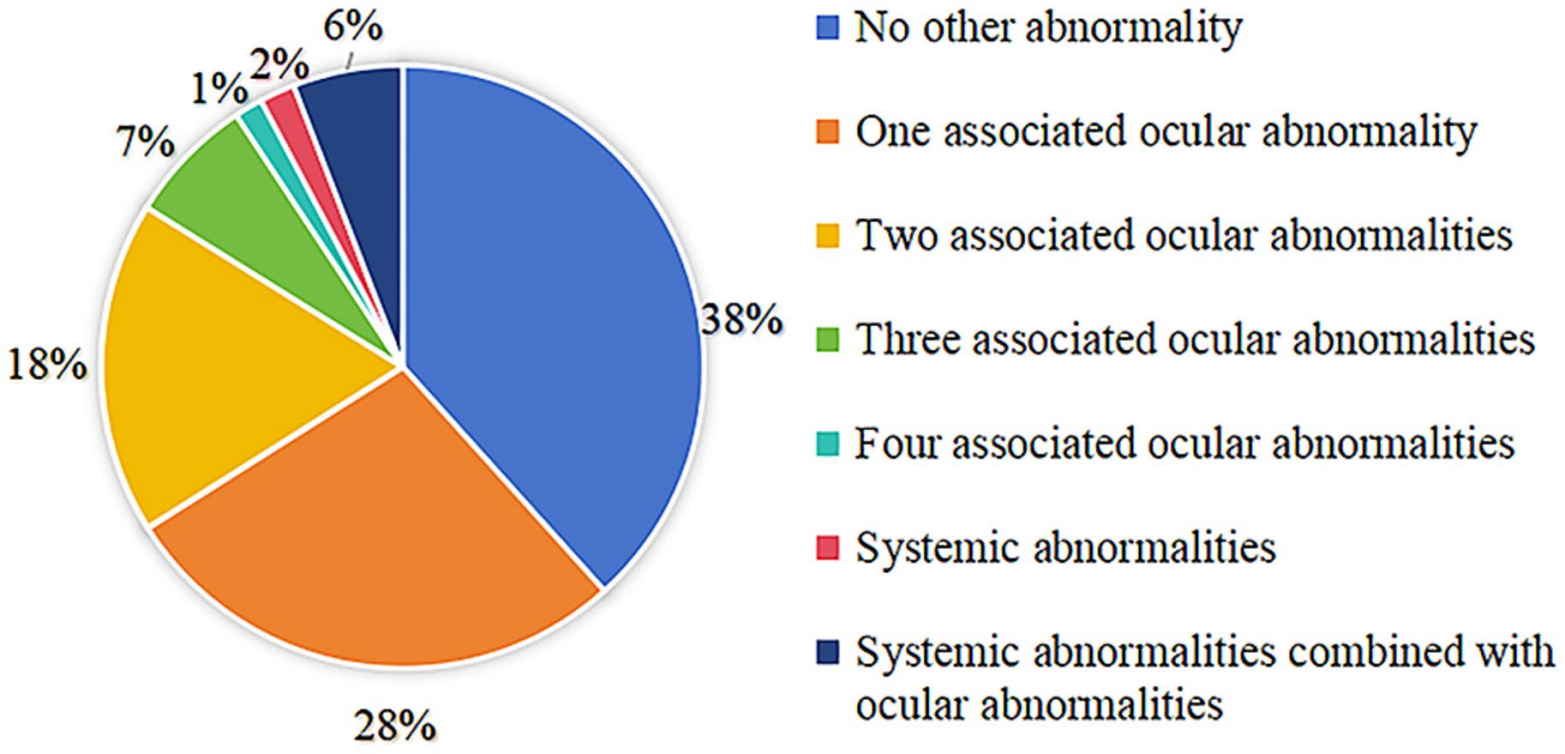
Distribution of patients with other ocular abnormalities and systemic abnormalities.

In total, 202 patients (38.33%) had no history of ocular or other systemic abnormalities. The distribution of patients with other ocular or systemic abnormalities is shown in Figure 5. There were 293 patients with combined ocular abnormalities, and 40 patients with combined systemic abnormalities. The details of the associated abnormalities are presented in Table 4. Amblyopia was the most common ocular abnormality, prevalent in 23.34% (123/527), followed by nystagmus (15.56%, 82/527), ametropia (9.11%, 48/527), exotropia (8.73%, 46/527), and persistent hyperplastic primary vitreous (PHPV) (7.97%, 42/527). In addition to ocular abnormalities, congenital heart and central nervous system diseases such as cerebral palsy, hypoxic-ischaemic encephalopathy, and hydrocephalus were also found to be associated with CC.

**Table 4:** The proportion of ocular abnormalities.

Associated abnormality	Number of patients (n)	Proportion (%)	95% CI
Amblyopia	123	23.34%	[19.92, 27.14%]
Nystagmus	82	15.56%	[12.70, 18.92%]
Refractive error	48	9.11%	[6.93, 11.88%]
Exotropia	46	8.73%	[6.60, 11.46%]
PHPV	42	7.97%	[5.94, 10.61%]

PHPV, persistent hyperplastic primary vitreous; CI, confidence interval.

## 4 Discussion

This large-scale, 16-year study elucidates the unique epidemiological and clinical characteristics of CC in Northwest China, a region burdened by healthcare disparities and delayed diagnosis. Our data reveal that CC constitutes a significant pediatric ophthalmic burden, with a pronounced rural predominance (61.29% of cases) and delayed surgical intervention in rural populations. Encouragingly, the median surgical age demonstrated a progressive decline over the study period, paralleling national efforts to improve neonatal screening. Critically, we identified dynamic AL growth patterns characterized by rapid elongation before age 2 years followed by stabilization after 6 years, which were further modulated by sex and laterality. These findings not only establish a foundation growth trajectory for a pediatric population in Northwest China, but also provide actionable targets for optimizing surgical timing and refractive rehabilitation in resource-limited settings.

Most CCs are treatable. However, although rare, CC is one of the most important causes of severe vision loss and is responsible for 5–20% cases of pediatric blindness worldwide (6–8). CC accounts for 2.39% of all cataract in-patients in a large hospital in Southern China (9). In our study, the proportion of CC among all cataract in-patients is higher than that previously reported (data not shown). This may be because Xijing Hospital, a tertiary referral center in Northwest China located in Xi’an (the region’s transportation hub), serves as a main centralized destination for CC patients under the province’s hierarchical healthcare system.

In the current study, rural patients constituted 61.29% of hospital admissions for CC, reflecting severe healthcare disparities in Northwest China. CC is 10 times more prevalent in low-income economies than in high-income economies (10). The median age at surgery of our entire CC populations was 34[5,72] months, which is greater than that reported in other studies (9, 11). Additionally, the median age at surgery was significantly higher in rural patients than in urban patients. This delay in surgery may be due to the relatively poor medical accessibility, social economic status and infrastructure differences in Northwest China. Many countries have established CC screening systems to identify children with CC at an early age (12). In China, although there is no national screening or follow-up system for CC, binocular vision screening instruments, the pupil red light reflex, and other methods have been used in some hospitals and cities. 61.29% rural patient predominance signals critical gaps in primary eye care, requiring mobile screening expansion in Northwest China. In the current study, most patients with CC were aged 2–6 years, whereas a minority were aged 12–18 years. Median surgical age differs significantly across hospitalization year subgroups, showing a slightly downward trend throughout the study period from 2008 to 2023. This trend may indicate an awareness of the importance of screening for and early treatment of eye diseases, meanwhile, this trend is partly attributed to changes in medical insurance policies, such as the increase in reimbursement rates under the New Rural Cooperative Medical Scheme. To bridge disparities in healthcare access, socioeconomic status, and infrastructure between Northwest China and other regions, concrete measures should be implemented, such as integrating CC screening into China’s National Newborn Disease Screening Program, subsidizing travel costs for low-income families through Provincial Critical Illness Funds, shifting diagnostic tasks to primary care.

Although various thresholds for age at surgery have been documented in the literature, ranging from 10 days to 5 years (13, 14), those who undergo surgery at or before the age of 9 months were 3.8 times more likely to progress to glaucoma than those who undergo surgery after the age of 9 months (15, 16). Although earlier removal of the opacified lens to allow light stimulation of the retina is more beneficial for promoting the normal development of visual pathways, the relationship between surgical timing and ocular development must be carefully considered, as premature intervention may be associated with an increased risk of complications such as glaucoma. Additionally, for preterm infants and low-birth-weight neonates, systemic anesthesia risks must be balanced against potential visual benefits. Furthermore, the northwest region is also facing the problem of uneven distribution of medical resources, and the diagnostic capabilities of primary care physicians in identifying CC and the referral systems require further improvement. Socioeconomic factors, parental education levels, the implementation of community-based neonatal eye screening programs, training of primary healthcare workers, and the availability of long-term postoperative rehabilitation resources, such as corrective glasses and contact lenses, may all influence the surgical timing for affected children. In our study, the median age of the CC patients who underwent cataract extraction surgery without IOL implantation was 5[3,11] months, which was different than the recommended age of 9 months. Although we did not investigate the prevalence and risk factors associated with secondary glaucoma post-CC surgery, future long-term follow-up studies are being conducted to focus on exploring the most appropriate surgical age to reduce the incidence of secondary glaucoma after CC surgery.

The AL is a crucial indicator of eyeball development and a vital contributor to surgical timing and IOL power calculation in patients with CC (17). The benefit of IOL implantation in CC with respect to visual prognosis, binocular function, ocular alignment, complications, and medical costs has been debated (18–20). Patients with CC who receive IOL implantation at a very young age experience a combination of greater prediction error (21), future myopic shift (22), unmatched IOL size (23), and a higher frequency of postoperative complications (24). The current cross-sectional study found a logarithmic correlation between AL and age at surgery in patients with CC, with a faster growing trend before the age of 24 months and a plateau thereafter. This indicated that AL increased more significantly in younger patients. This result is consistent with previous reports (25, 26). The current belief is that delaying IOL implantation until older age, after most AL growth has occurred, is conducive to selecting a more appropriate IOL power for implantation (17, 27). Based on the AL growth rate, performing IOL implantation after the age of 2 years is a relatively better option. In our study, the median age for IOL implantation during cataract surgery was 72[42,120] months, greater than that reported in other studies (26). This is due to the inclusion of CC patients aged >18 years.

Eyes with CC have different biometric measurements from normal eyes. AL growth can be influenced by various factors, including age at surgery, aphakia, pseudophakia, cataract laterality, and sex (17, 28). The current study found that in patients with bilateral CC, the AL of male patients aged 6M–2Y, 2Y–6Y was significantly longer than that of their female counterparts. This indicates a close correlation between AL and head size, as boys have a larger head circumference than girls (29). Sex-related differences in AL among bilateral CC patients further complicate IOL selection, suggesting potential sex-specific adjustments may be needed in IOL power formulas. This divergence may stem from hormonal or genetic influences on ocular development, warranting further investigation. Notably, the absence of sex-based differences in unilateral CC implies that asymmetric deprivation may override intrinsic growth patterns, necessitating individualized surgical planning.

Further, we found that AL was related to laterality. The significant AL differences between affected and fellow eyes in unilateral CC patients aged 6M–2Y likely reflect asymmetric visual deprivation effects. This is consistent with previous results (30). These disparities emphasize the need for precise IOL power calculations to address anisometropia, a key risk factor for amblyopia. For instance, in unilateral cases, overcorrection of the affected eye may reduce anisometropic disparity, though this must be balanced against future myopic shifts due to continued AL growth. However, Zhang et al. (31) reported a significantly shorter AL in the affected eye than in the fellow normal eye in patients with CC aged <24 months. All these findings reveal that AL in patients with unilateral CC may not only be affected by age, but also by other factors that require further study. The high proportion of unilateral CC (33.78%) underscores the challenge of managing unilateral aphakia. In our study, AL discrepancies may necessitate delayed IOL implantation to mitigate refractive surprises. For patients aged<2 years, leaving the eye aphakic with rigorous optical correction such as silicone contact lenses could provide flexibility until AL stabilizes. However, in some remote areas of Northwest China, where access to specialized contact lenses is limited or patients face inaccessibility to follow-up care, earlier IOL implantation in severe unilateral cases may be prioritized to reduce long-term dependence on scarce resources.

The findings in our study advocate for regionally adapted clinical guidelines. For example, AL growth curves specific to Northwest China could refine IOL power formulas like Hoffer Qped. Additionally, targeted training for rural ophthalmologists in AL measurement and IOL calculation may reduce postoperative refractive errors, which are particularly detrimental in regions with limited follow-up capacity. AL variations in CC patients are not merely anatomical metrics but critical determinants of surgical timing, IOL selection, and postoperative rehabilitation. Integrating these insights into context-specific protocols, particularly in resource-constrained settings, is essential to optimize visual outcomes while addressing systemic inequities in pediatric ophthalmic care.

Ocular or systemic abnormalities significantly affect the prognosis of CC. Preoperative nystagmus indicates impending amblyopia or other types of visual impairment (32). We further analyzed the postoperative best corrected visual acuity of patients with CC with and without nystagmus but found no significant difference between them (data not shown). This may be because the limited observation period and absence of extended follow-up duration in this study, which could have constrained the comprehensive assessment of long-term clinical outcomes. Further investigations with prolonged observational periods are warranted to elucidate the prognostic trajectory of CC patients. The high proportion of ocular comorbidities and systemic abnormalities in CC patients demands individualized clinical strategies. Surgically, PHPV demands preoperative B-ultrasound scan to evaluate retro-lental fibrovascular complexity, often requiring combined anterior vitrectomy (33), while coexisting congenital heart disease mandates multidisciplinary risk stratification for anesthesia. Postoperatively, amblyopia and nystagmus require aggressive postoperative refractive correction immediately to maximize visual rehabilitation potential; cerebral palsy and hypoxic-ischaemic encephalopathy impedes compliance with optical correction and patching regimens, requiring multidisciplinary neuro-rehabilitation integration. Concurrent exotropia may indicate sensory strabismus, prompting consideration of combined or staged strabismus surgery to restore binocularity. These findings underscore that comorbidity-driven protocols, not standardized approaches, optimize functional outcomes in this complex population.

Our results should be interpreted with caution in consideration of the following limitations. Firstly, this study is inherently limited by the single-center design at a tertiary referral hospital in Northwest China, which may introduce selection bias toward complex CC cases under the region’s hierarchical medical system. Further, the inadequate healthcare systems, malnutrition, and higher rates of perinatal infections in Northwest China may have introduced a bias in the proportion of CC and age at surgery. Thirdly, as a cross-sectional study, our current design focused exclusively on preoperative AL patterns and their epidemiological correlates. While we recognize the critical importance of postoperative outcomes, longitudinal assessment falls beyond the scope of this cross-sectional investigation. The impact of axial length variations on postoperative rehabilitation requires further longitudinal verification. Despite these limitations, the results of this study described the overall epidemiological characteristics, preoperative AL, and proportion of associated ocular diseases in Northwest China. Future multi-center clinical studies that focus on CC should be conducted in Northwest China and long-term follow-up studies are required to better understand the prognosis of patients with CC.

## 5 Conclusion

In conclusion, this study described the prevalence, sex distribution, age at surgery, distribution of AL, laterality, and associated ocular abnormalities in patients with CC in Northwest China. Our study provides a theoretical basis for the further prevention and treatment of CC and for increasing the proportion of patients undergoing surgery at a critical time for visual development in Northwest China.

## Data Availability

The original contributions presented in the study are included in the article/Supplementary material, further inquiries can be directed to the corresponding authors.
