# Supplementary material for: Epidemiological characteristics, distribution of axial length, and prevalence of ocular abnormalities associated with congenital cataract in Northwest China: a 16-year cross-sectional study (2008–2023)
Source: Front Med (Lausanne). 2025 Sep 5;12:1617601. doi: 10.3389/fmed.2025.1617601 (PMC12446306; doi:10.3389/fmed.2025.1617601)
Supplement: Supplementary file 1 [file Table_1.DOCX]

Table S1.Demographic characteristics of all participants.

| **Demographic variable** | ****Subgroups**** | ****N**** |
| --- | --- | --- |
| Gender | Female | 247  280 |
|  | Male |  |
| Regional distribution | Rural | 323  204 |
|  | Urban |  |
| laterality | Bilateral | 349  178 |
|  | Unilateral |  |
| Surgical strategy | Without IOL implantation | 256  271 |
|  | With IOL implantation |  |

N=number of patients

Table S2.The results of the Kolmogorov-Smirnov tests.

| ****Variable**** | ****D-statistic**** | ****p-value**** | ****Normality Conclusion**** |
| --- | --- | --- | --- |
| Axial length (mm) | 0.072 | 0.192 | Not rejected (Normal) |
| Age at surgery (months) | 0.240 | ****<0.001**** | Rejected (Non-normal) |
| Rural patients’ age at surgery (months) | 0.225 | ****<0.001**** | Rejected (Non-normal) |
| Urban patients’ age at surgery (months) | 0.263 | ****<0.001**** | Rejected (Non-normal) |
